# Differences in cardiovascular risk and health-related quality of life in COPD patients according to clinical phenotype

**DOI:** 10.1038/s41598-024-60406-x

**Published:** 2024-04-27

**Authors:** Ana Muñoz Montiel, Pedro Ruiz-Esteban, Adolfo Doménech Del Río, Pedro Valdivielso, Miguel Ángel Sánchez Chaparro, Casilda Olveira

**Affiliations:** 1 Pulmonology Service. Monographic COPD Office, Regional University Hospital of Malaga, University of Malaga, Malaga, Spain; 2 Nephrology Department, The Biomedical Research Institute of Malaga (IBIMA-Plataforma BIONAND), Regional University Hospital of Malaga, University of Malaga, RICORS2040 (RD21/0005/0012), Malaga, Spain; 3 Laboratory of Lipids and Atherosclerosis, Medico-Sanitarias Research Center (IBIMA), University of Malaga, Malaga, Spain; 4 Internal Medicine, University Hospital Virgen de La Victoria, Department of Medicine and Dermatology and Biomedical Research Institute of Malaga (IBIMA), Platform Bionand, University of Malaga, Malaga, Spain; 5 Pulmonology Service, Regional University Hospital of Malaga, Department of Medicine and Dermatology and Biomedical Research Institute of Malaga (IBIMA), Platform Bionand, University of Malaga, Malaga, Spain

**Keywords:** COPD, Health-related quality of life, Cardiovascular risk, Phenotype, COPD exacerbation, Diseases, Health care, Medical research, Risk factors

## Abstract

Chronic obstructive pulmonary disease (COPD) has a high prevalence and a major impact on health-related quality of life (HRQL). COPD exacerbations are an important cause of morbidity and mortality, affecting cardiovascular risk, and are associated with poorer health status. The aim of this study was to assess the association between cardiovascular risk (CVR) and HRQL, according to exacerbator or non-exacerbator phenotype. We undertook a cross-sectional, observational, descriptive study of 107 patients with COPD. Patients with two or more moderate exacerbations or one severe exacerbation in the previous year were considered as exacerbators. The CVR was calculated with the Framingham scale and SCORE (Systematic Coronary Risk Evaluation) and the HRQL was assessed with the generic questionnaire Short Form-36 Health Survey (SF-36), the St George Respiratory Questionnaire (SGRQ) and the COPD Assessment Test (CAT). Statistical analysis was done with SPSS version 26.0 for Windows. The SF-36 and the SGRQ showed lower values for the exacerbator phenotype, indicating a poorer quality of life. The CAT questionnaire showed values above 10 for the exacerbator phenotype, and lower values in the non-exacerbator group. After categorizing the sample according to their median age (65 years), we found a greater deterioration in HRQL in patients under 65 years of age according to the SF-36, the SGRQ and the CAT. We also detected differences in HRQL between non-exacerbator patients with a high CVR according to the Framingham (≥ 20%) and SCORE (≥ 5%) scales compared to those without this risk. A tendency towards worse HRQL was observed in non-exacerbator patients with a high CVR, which was statistically significant for the SGRQ impact domain on the SCORE scale. The CAT also showed a worse quality of life in non-exacerbator patients with a high CVR, which was significant in the Framingham model (Framingham high risk 8.41 vs non-high risk 6.05, p < 0.01). These differences were not observed in exacerbator patients. Our findings confirm that a high CVR influences HRQL in patients with COPD, especially in non-exacerbator patients with a high CVR, measured according to the SGRQ and the CAT.

## Introduction

Chronic obstructive pulmonary disease (COPD) is a disease state characterised by the presence of persistent respiratory symptoms such as dyspnoea, cough and/or expectoration, exacerbations, and chronic airflow limitation. COPD is secondary to chronic exposure to tobacco smoke as the main causative agent, among other inhaled substances. Moreover, it is a chronic systemic inflammatory disease even in those patients who no longer smoke, leading to an increase in inflammatory cells in the airway, greater oxidative stress and, consequently, a higher prevalence of other chronic inflammatory diseases. The most prevalent comorbidities in COPD (hypertension, dyslipidaemia, diabetes mellitus) and its complications (heart failure, atrial fibrillation, stroke, retinopathy, neuropathy and ischaemic heart disease) condition quality of life^1–5^. Thus, the estimation of cardiovascular risk (CVR), which encompasses the main vascular risk factors, is an important indicator of the potential effects of these comorbidities in the development of cardiovascular disease and the best tool to establish priorities in cardiovascular prevention^6^.

Health-related quality of life (HRQL) is important in itself and represents one of the main aims of all health interventions^7,8^. Quality of life questionnaires are designed to provide standard measures of health impairment and must evaluate the gap between the current disease-related HRQL and the desirable lifestyle. There are both generic and specific quality-of-life questionnaires^9^. The SF-36 questionnaire has proved its validity in the evaluation of COPD patients and offers a good correlation with the baseline dyspnoea index, allowing assessment of the quality of life of COPD patients in relation to previous admissions and comorbidities^10^. Among the COPD-specific questionnaires, the SGRQ allows us to quantify the impact of airway diseases on the health condition and perceived well-being of respiratory patients^11^. It is sensitive to changes in disease progression and therapeutic response. The CAT also provides a quick, self-administered and simple way to measure the impact of COPD on HRQL^12^. Exacerbations in COPD and their severity affect many different factors related with the HRQL of these patients. As widely reported, patients who experience frequent exacerbations have a worse HRQL in comparison to patients who experience fewer exacerbations (< 2/year)^13,14^. Indeed, improving the HRQL is associated with a lower risk of exacerbations^15^.

Both comorbidities and exacerbations in COPD patients have a direct impact on the HRQL. In addition, they are an important cause of hospital morbidity and mortality and increase the risk of cardiovascular disease, including myocardial infarction and stroke^16–18^. A few studies have analysed the association between CVR and HRQL: a cohort of patients with hepatitis C was studied, finding a relation between patients with a high CVR and worse HRQL, though not significantly so^19^. Another study analysed mental disorders and CVR and their association with HRQL, where a subgroup with depressive disorders presented a higher CVR and worse HRQL results^20^. Very few studies in patients with COPD have found an association between CVR and HRQL, either globally or according to phenotype, associating a worsening HRQL in exacerbator patients due to the exacerbations themselves and hospital admissions rather than to other reasons, such as a high CVR.

A comparative analysis of the estimated CVR in COPD patients according to their phenotype (exacerbator or non-exacerbator) and its effect on HRQL is relevant as it may contribute to orienting different preventive strategies and, thus, a more efficient preventive intervention.

Our aim in this study was to establish whether there exists a relation between CVR and HRQL among patients with COPD according to exacerbator phenotype and whether differences exist between the two groups.

## Material and methods

### Study design

Observational, cross-sectional, descriptive, random study of 107 patients diagnosed with COPD and referred to the monographic COPD office at the Regional University Hospital of Malaga, Spain.

### Inclusion and exclusion criteria

Inclusion: Age between 40 and 75 years, history of smoking greater > 10 packs/year, spirometry with post-bronchodilator FEV_1_/FVC ratio < 70%, post-bronchodilator FEV_1_ < 80%, clinical stability during the eight weeks prior to inclusion in the study (according to information from the patient and electronic medical history), ability to answer the questionnaires and availability to attend for the supplementary tests.

Exclusion: presence of concomitant pulmonary disease (such as pulmonary tuberculosis), malignant disease, recent cardiovascular event (within previous 6 months), presence of COPD exacerbation within 8 weeks of the study (in this case inclusion was postponed until 8 weeks had passed), patients who had previously participated in another study or clinical trial, and those who did not sign the informed consent.

The study was undertaken in accordance with the norms of good clinical practice and the ethical concepts of the Declaration of Helsinki (21). All the patients signed the informed consent and the study was accepted by the provincial Research Ethics Committee on 20 December 2013 with the code 6/2020 PI 10.

### Data collection

A detailed medical record was elaborated with the following variables:

- Demographic data: age, gender and marital status
- Smoking habits
- Personal history: Hypertension, dyslipidaemia, diabetes, cardiovascular disease (CVD), atrial fibrillation
- Charlson comorbidity index^22^. Comorbidity was defined as any additional entity (disease, health condition) that has existed or may occur during the clinical course of a patient with a disease^23^
- Treatments, including continuous home supplemental oxygen, long-acting beta_2_-agonist, long-acting muscarinic antagonist, or inhaled corticosteroids.
- The Modified Medical Research Council (mMRC) dyspnoea scale, according to patient-reported data and data in the electronic medical record of the Andalusian Health Service (SAS). Based on these data, it was established whether the patient had an exacerbator or non-exacerbator phenotype. Exacerbators were considered patients with COPD who had had two or more exacerbations in the previous year, moderate (in which their usual treatment had been modified by adding antibiotics and/or corticosteroids) or severe (with hospitalisation). These exacerbations had to be at least 8 weeks apart from the conclusion of previous exacerbation treatment to avoid mistaking them for treatment failures^1^.

Physical examination:

- Height and weight measured by stadiometer and scales (*SECA 665, Seca*), from which the body mass index (BMI) was calculated
- Abdominal circumference, with a normal tape measure
- Two blood pressure (BP) readings from the same arm employing a validated automatic device (OMROM M4-I, Omron Electronics, Hoofddorp, Netherlands) with an interval of 1–2 min between readings. The BP was measured with a manual sphygmomanometer with participants resting for at least five minutes. Three recordings were taken and the average of the second and third readings was used in statistical analyses^25^.

Analytical determinations:

Laboratory measurements were analysed in the Regional University Hospital of Malaga, obtaining a blood sample after 12-h fasting.

- Complete blood count (haemoglobin, haematocrit, white blood cells, eosinophilic and differential blood count), coagulation, glucose, urea, creatinine, uric acid, sodium, potassium, stable glycosylated haemoglobin (HbA_1c_), cholesterol, HDL-cholesterol, LDL-cholesterol, triglycerides, transaminases, total proteins, serum albumin, immunoglobulin E, and alpha-1-antitrypsin.

Estimation of CVR.

CVR was estimated from the following models.

- Framingham global CVR score, with a Framingham ≥ 20% considered a high CVR^26^,
- European SCORE risk chart for low-risk countries^27^, following the previously published procedure and considering a SCORE ≥ 5% as a high CVR.

Respiratory function:

- Spirometry and bronchodilator test. All the patients underwent a spirometry with a bronchodilator test following the guidelines of the Spanish Society of Pneumology and Thoracic Surgery (SEPAR) for this technique^20^ with a JAEGER (OXICOM) pneumotachograph.
- Static volumes: Measured using a JAEGER (OXICOM) plethysmograph, determining total lung capacity, residual volume and inspiratory capacity following the SEPAR Procedures Manual^28–30^.
- A 6-min walk test (6MWT): recording metres, O_2_ saturation at the beginning and at the end of the test, initial and final heart rate, and the initial and final Borg Scale of dyspnoea perceived by the patient before and after exercise^31^. The 6MWT was carried out following the 2002 guidelines of the American Thoracic Society^32^ and always performed by the same nurse.
- BODE used as a prognostic tool in COPD, which combines FEV_1_, distance travelled in the 6MWT, the mMRC Dyspnoea Scale value and BMI parameters^33^.

Quality of life questionnaires:

- The Short Form-36 Health Survey (SF-36). This is a generic questionnaire composed of 36 items, examining eight dimensions of health status: physical functioning, social functioning, role limitations due to physical problems, role limitations due to emotional problems, mental health, vitality, pain and general health perception. The score ranges from 0 to 100 for each dimension, and does not allow an overall score to be calculated^10,34^.
- The Saint George Respiratory Questionnaire (SGRQ), a specific tool to assess HRQL and quantify the impact of airway diseases on health status and perceived wellbeing of respiratory patients^11,35^. It is composed of 50 items, distributed in 3 dimensions: symptoms, activity and impact. The score ranges from 0 to 100, with 100 being the maximum change in HRQL; the minimum change considered clinically relevant has been set at 4 units^36^.
- The Chronic Obstructive Pulmonary Disease Assessment Test (CAT), developed to assess the impact of COPD in HRQL simply and quickly, is a specific and self-administered questionnaire. Each item has 5 response options. The final score of the scale is equal to the sum of the scores for each item, with a range from 0 to 40, where a higher score indicates a greater impact of COPD on the patient quality of life^12,37–39^. We considered a score > 10 a worse quality of life. We established a time of 8 weeks between the onset of exacerbation and HRQL testing to ensure that the patient was in a stable phase.

### Statistical analysis

Information was processed in a database developed specifically for this purpose. Statistical analysis was performed using SPSS software version 26.0 for Windows (IBM Corp., Armonk, NY, USA). Quantitative variables are expressed as the mean (measures of centralisation) ± standard deviation (measures of variability). In the case of qualitative variables, relative and absolute frequencies are used. Normality analysis was performed for all variables using the Kolmogorov–Smirnov test. For comparison between quantitative variables between groups, parametric (Student T-test) or non-parametric (Mann–Whitney U test) tests were used, as appropriate. Comparison between groups of qualitative variables was performed using the Chi-Square test or Fisher test, depending on whether or not the data followed a normal distribution. Univariate and multivariate logistic regression analyses were performed to assess the association between scores and the endpoint variables after adjustment for confounders. Variable associations were assessed by estimating the Pearson or Spearman correlation coefficient. Values of less than 0.05 (*p* < 0.05) for two tails were considered statistically significant differences.

## Results

**Figure 1:**
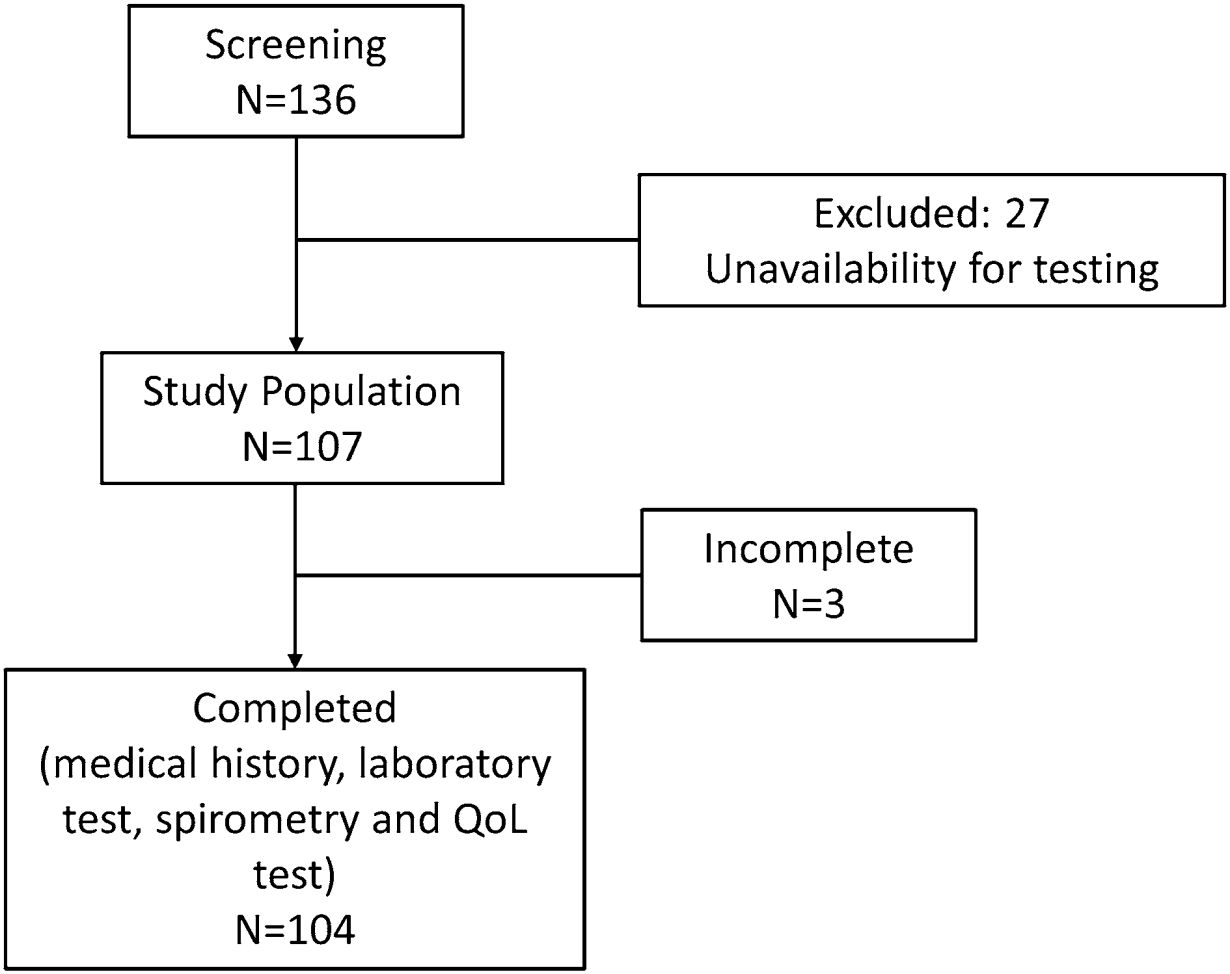
Patient participation and selection flowchart.

Of the 136 patients initially evaluated, 27 were excluded due to unavailability to perform the necessary examinations and another two were excluded because they did not meet the inclusion criteria. Out of the remaining 107 patients whose clinical history, laboratory tests and respiratory function tests were completed, the quality-of-life tests could not be performed in 3 patients for various reasons (see Fig. 1). Table 1 presents the general characteristics of the population by phenotype. Most patients (73.8%) were men, and the average age was 63.1 ± 6.6 years. The most frequent spirometry pattern was severe obstruction according to GOLD^1^, with a substantial level of air-trapping. Significant differences were found in diastolic blood pressure, which was higher in the non-exacerbator group (87.2 ± 12.4 vs 81.8 ± 11.5 mmHg; *p* = 0.022), and more so in the patients under 65 years of age. The average BODE Index^25^ was within the moderate stage, showing significant differences between the exacerbator and non-exacerbator groups (3.4 ± 1.6 vs. 2.6 ± 1.5; *p* = 0.018); 23.4% of the patients had CVD, 16.8% had diabetes mellitus, 37.4% had dyslipidaemia, and 40.2% had the metabolic syndrome. One in five had some comorbidity in addition to COPD and 15% had more than one comorbidity. In the Framingham and SCORE models, more than 50% were within the high or very high cardiovascular risk range^18,19^.

**Table 1:** General characteristics of the study population according to phenotype.

	Total N = 107	Exacerbator n = 50	Non-exacerbator n = 57	P
Sex (male) %	79 (73.8)	38 (76.0)	41 (71.9)	0.663
Age (years)	63.1 ± 6.6	63.4 ± 5.7	62.9 ± 7.3	0.771
Smoker %	34 (31.8)	16 (32.0)	18 (31.6)	0.963
Dyspnoea mMRC	1.81 ± 0.60	1.92 ± 0.57	1.72 ± 0.62	0.054
FVC (ml) post	2308 ± 722.0	2211 ± 605.7	2393 ± 806.2	0.186
FVC (%) post	**58.5 ± 14.1**	**55.3** ± **12.9**	**61.3** ± **14.6**	**0.027**
FEV1 (ml) post	1244 ± 473.4	1213 ± 400.3	1271 ± 531	0.520
FEV1 (%) post	43.9 ± 14.1	41.8 ± 11.9	45.7 ± 15.7	0.153
FEV1/FVC (%)	52.1 ± 10.7	53.6 ± 10.4	50.8 ± 10.9	0.180
RV/TLC (%)	64.2 ± 12.8	64.8 ± 13.1	63.7 ± 12.6	0.655
6MWT (m)	414.3 ± 84.2	397.9 ± 94.3	429.4 ± 71.3	0.058
6MWT (%)	90.9 ± 19.6	87.2 ± 22.2	94.5 ± 16.3	0.150
Basal HR (bpm)	82.4 ± 13.7	83.8 ± 15.3	81.1 ± 12.1	0.322
Final HR (bpm)	105.0 ± 18.3	105.6 ± 19.4	104.5 ± 17.4	0.771
Basal O2 sat	95.2 ± 1.9	95.1 ± 2.1	95.3 ± 1.7	0.744
Final O2 sat	91.0 ± 6.5	90.1 ± 4.8	91.9 ± 7.7	0.409
BODE Index	**3.0 ± 1.6**	**3.4** ± **1.6**	**2.6** ± **1.5**	**0.018**
BMI (kg/m2)	27.9 ± 5.4	28.8 ± 7.5	27.8 ± 4.9	0.549
SBP (mmHg)	141.9 ± 20.2	138.5 ± 21.7	145.1 ± 18.3	0.091
DBP (mmHg)	**84.6 ± 12.2**	**81.8** ± **11.5**	**87.2** ± **12.4**	**0.022**
Comorbidities (%)				
CVD	25 (23.4)	11 (22.0)	14 (24.6)	0.755
AF	10 (9.3)	7 (14.0)	3 (5.3)	0.121
HBP	66 (61.7)	32 (64.0)	34 (59.6)	0.644
DM	18 (16.8)	11 (22.0)	7 (12.3)	0.180
DL	40 (37.4)	18 (36.0)	22 (38.6)	0.782
Chronic kidney disease	3 (2.8)	1 (2.0)	2 (3.5)	0.637
Metabolic syndrome	43 (40.2)	21 (42.0)	22 (38.6)	0.720
CVR:				
Framingham	26.4 ± 16.5	27.2 ± 17.5	25.7 ± 15.7	0.591
SCORE	7.9 ± 10.5	8.4 ± 12.4	7.5 ± 8.7	0.549
QRisk	20.8 ± 11.7	22.4 ± 12.6	19.3 ± 10.7	0.173
Regicor	6.3 ± 3.4	6.5 ± 3.6	6.2 ± 3.2	0.650

Data are expressed as mean ± standard deviation.

AF, atrial fibrillation; BMI, body mass index; CVD, cardiovascular disease; CVR, cardiovascular risk; DBP, diastolic blood pressure; DM, diabetes mellitus; DL, dyslipidaemia; FVC, forced vital capacity; FEV1, forced expiratory volume in 1 s; HBP, high blood pressure; HR, heart rate; post, post-bronchodilator; SBP, systolic blood pressure; TLC, total lung capacity; RV, residual volume; 6MWT, 6-min-walk test.

In bold, differences that reach statistical significance (p < 0.05).

**Figure 2:**
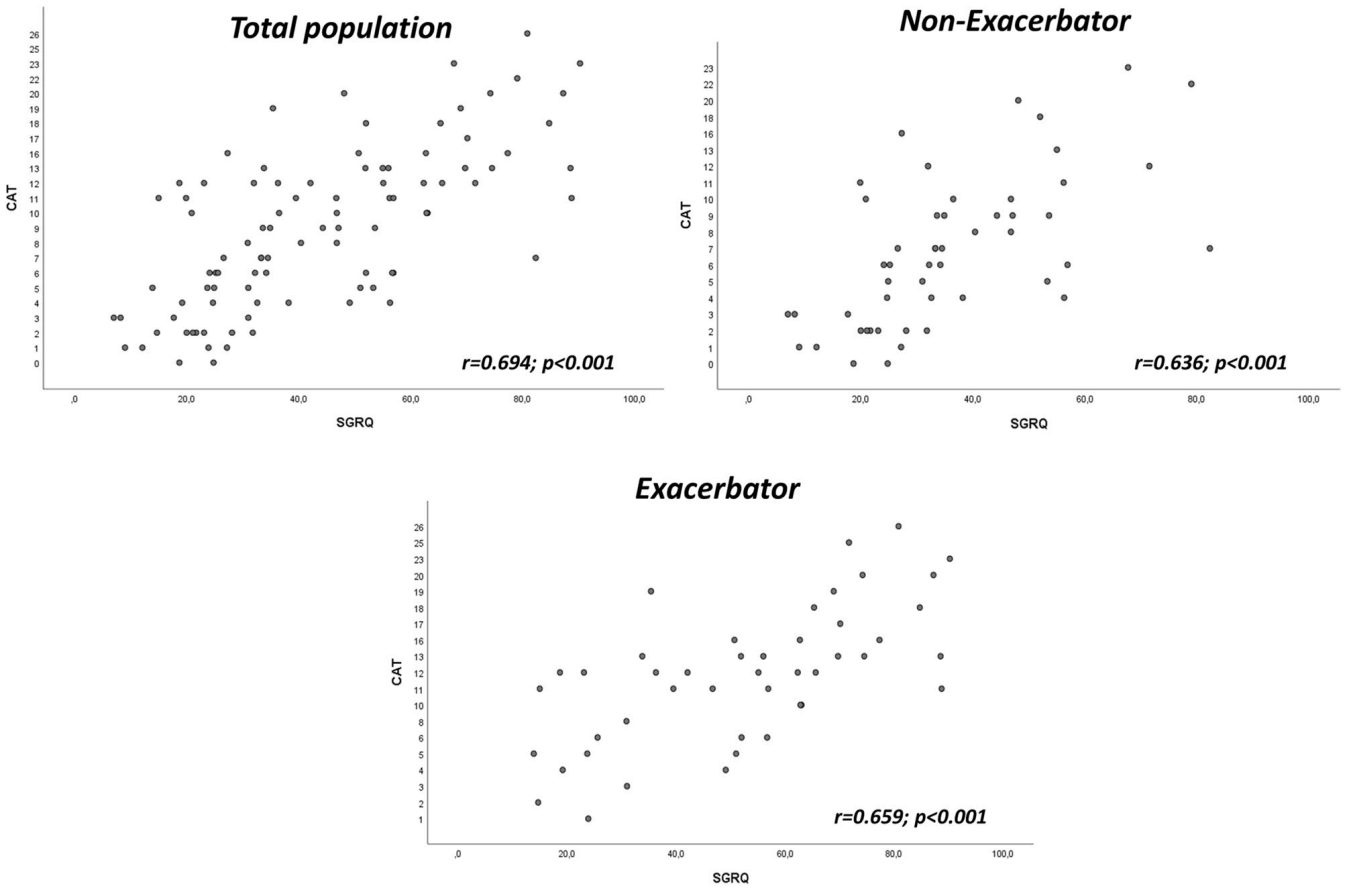
correlation between CAT (COPD Assessment Test) and SGRQ (Saint George Respiratory Questionnaire).

The generic SF-36 questionnaire showed lower values for the exacerbator phenotype (worse HRQL), reaching statistical significance in the scores for bodily pain (57.5 ± 35.2 vs. 73.7 ± 28.9; *p* = 0.033), physical functioning (41.4 ± 25.3 vs. 60.3 ± 24.7; *p* < 0.001), general health perception (31.4 ± 17.2 vs. 44.0 ± 20.7; *p* = 0.004) and vitality (44.1 ± 26.2 vs. 56.3 ± 24.2; *p* = 0.017). In the respiratory disease-specific questionnaires, the SGRQ also showed a worse HRQL for patients with the exacerbator phenotype, reaching statistical significance in all scores. The CAT questionnaire showed values above 10 in the exacerbator phenotype, indicating the need to change treatment or establish new therapeutic measures; these values were significantly higher than in the non-exacerbator phenotype (11.8 ± 6.1 vs. 7.4 ± 5.5; *p* < 0.001) (Table 2). A good correlation was found between the values of the specific questionnaires (SGRQ and CAT) for both phenotypes, as shown in Fig. 2. Table 3 shows the differences in the values of the quality-of-life questionnaires (SF-36, SGRQ and CAT) between patients who did or did not require hospital admission due to exacerbation of their COPD in the year prior to the study. A worse quality of life was found in those patients who had to be admitted, significantly so in the SGRQ scores for activity (70.7 ± 25.5 vs. 55.6 ± 24.2; *p* = 0.016) and symptoms (59.9 ± 23.8 vs. 45.2 ± 25.0) and in the CAT questionnaire (13.4 ± 7.8 vs. 8.6 ± 5.4; *p* = 0.017). When analysing the subject population according to the median age (65 years), a significant deterioration in HRQL was observed in the exacerbator phenotype in those patients under 65 years of age, where there was a greater worsening in the scores of bodily pain (56.5 ± 33.9 vs. 81.8 ± 21.1; *p* = 0.006), physical functioning (7.7 ± 25.9 vs. 59.8 ± 23.8; *p* = 0.004) and general health perception (30.2 ± 18.4 vs. 44.0 ± 23.3; *p* = 0.047) in the SF-36 questionnaire. In the SGRQ, the deterioration was greater in the scores for activity (69.8 ± 26.9 vs. 50.5 ± 23.0; *p* = 0.006) and impact (45.9 ± 24.5 vs. 30.2 ± 18.2; *p* = 0023) for the exacerbator subgroup under 65 years of age and in the symptoms score (56.9 ± 21.5 vs. 35.1 ± 20.0; *p* = 0.001) in the exacerbators over 65 years of age. The CAT questionnaire was also significantly worse in the exacerbators under 65 years of age (12.6 ± 6.0 vs. 7.1 ± 5.9; *p* = 0.001) (Table 4). Logistic regression analysis adjusted for confounders (Table 5) showed that the exacerbator phenotype and COPD severity were risk factors associated with worse HRQL.

**Table 2:** Values of the quality of life questionnaires in the study population according to phenotype.

	Exacerbator n = 50	Non-exacerbator n = 57	p
SF-36 Health Change over Time	52.7 ± 27.7	61.3 ± 28.4	0.170
SF-36 Bodily Pain	**57.5 ± 35.2**	**73.7 ± 28.9**	**0.033**
SF-36 Physical Functioning	**41.4 ± 25.3**	**60.3 ± 24.7**	** < 0.001**
SF-36 Social Functioning	61.4 ± 32.0	71.8 ± 29.1	0.101
SF-36 Role Limitations Due to Physical Problems	47.9 ± 46.3	60.8 ± 39.1	0.185
SF-36 General Health Perceptions	**31.4 ± 17.2**	**44.0 ± 20.7**	**0.004**
SF-36 Role Limitations Due to Emotional Problems	66.0 ± 42.6	76.7 ± 37.9	0.140
SF-36 Mental Health	60.8 ± 23.4	66.6 ± 25.3	0.113
SF-36 Vitality	**44.1 ± 26.2**	**56.3 ± 24.2**	**0.017**
SGRQ Activity	**67.4 ± 24.7**	**50.7 ± 22.9**	**0.001**
SGRQ Impacts	**42.7 ± 22.8**	**28.6 ± 18.7**	**0.003**
SGRQ Symptoms	**57.6 ± 23.4**	**39.6 ± 22.5**	** < 0.001**
SGRQ Total	**51.5 ± 22.7**	**36.7 ± 17.5**	**0.001**
CAT	**11.8 ± 6.1**	**7.4 ± 5.5**	** < 0.001**

CAT, COPD assessment test; SF-36, short form-36 health survey; SGRQ, Saint George Respiratory Questionnaire; assessment test.

In bold, differences that reach statistical significance (p < 0.05).

**Table 3:** Differences in quality of life between patients admitted and not admitted in the year prior to inclusion in the study.

	Admitted n = 19	Not Admitted n = 88	p
SF-36 Physical functioning	41.6 ± 23.6	53.7 ± 26.9	0.057
SF-36 General Health Perception	31.1 ± 24.0	39.7 ± 18.8	0.065
SGRQ Activity	**70.7 ± 25.5**	**55.6 ± 24.2**	**0.016**
SGRQ Impacts	45.5 ± 25.3	32.8 ± 20.3	0.061
SGRQ Symtoms	**59.9 ± 23.8**	**45.2 ± 25.0**	**0.019**
SGRQ Total	52.5 ± 25.0	45.2 ± 24.0	0.092
CAT	**13.4 ± 7.8**	**8.6 ± 5.4**	**0.017**

Data are expressed as mean ± standard deviation.

CAT, COPD assessment test; SF-36, short form-36 health survey; SGRQ, Saint George Respiratory Questionnaire.

In bold, differences that reach statistical significance (p < 0.05).

**Table 4:** Quality of life categorised by median age (65 years) according to phenotype.

	≤ 65 years			> 65 years		
	Exacerbator N = 29	Non-exacerbator N = 28	p	Exacerbator N = 21	Non-exacerbator N = 29	p
SF-36 Health Change over Time	48.2 ± 28.0	65.0 ± 31.5	0.052	59.2 ± 26.6	58.0 ± 25.5	0.804
SF-36 Bodily Pain	**56.5 ± 33.9**	**81.8 ± 21.1**	**0.006**	59.1 ± 37.8	66.5 ± 33.1	0.651
SF-36 Physical Functioning	**37.7 ± 25.9**	**59.8 ± 23.8**	**0.004**	46.8 ± 24.1	60.7 ± 25.8	0.057
SF-36 Social Functioning	57.8 ± 32.0	69.6 ± 33.9	0.076	71.0 ± 30.4	73.8 ± 24.6	0.947
SF-36 General Health Perception	**30.2 ± 18.4**	**44.0 ± 23.3**	**0.047**	33.2 ± 15.4	43.9 ± 18.5	0.051
SF-36 Vitality	44.1 ± 24.2	56.6 ± 25.8	0.074	44.2 ± 29.6	56.1 ± 23.1	0.118
SGRQ Activity	**69.8 ± 26.9**	**50.5 ± 23.0**	**0.006**	**63.8 ± 21.3**	**50.8 ± 23.1**	**0.040**
SGRQ Impacts	**45.9 ± 24.5**	**30.2 ± 18.2**	**0.023**	38.1 ± 19.8	27.2 ± 19.3	0.067
SGRQ Symptoms	58.1 ± 24.9	44.5 ± 24.4	0.058	**56.9 ± 21.5**	**35.1 ± 20.0**	**0.001**
SGRQ Total	**53.4 ± 25.0**	**38.3 ± 17.9**	**0.031**	**48.8 ± 19.0**	**35.2 ± 17.3**	**0.011**
CAT	**12.6 ± 6.0**	**7.1 ± 5.9**	**0.001**	10.5 ± 6.2	7.7 ± 5.2	0.104

Data are expressed as mean ± standard deviation.

CAT, COPD Assessment Test; SF-36, Short Form-36 health survey; SGRQ, Saint George Respiratory Questionnaire.

In bold, differences that reach statistical significance (p < 0.05).

**Table 5:** Logistic regression model using as dependent variable having a CAT score equal to or higher than 10 and having an SGRQ score equal to or higher than 5.

	OR (95% CI)	*p*
CAT		
Exacerbator Phenotype	4.648 (1.832–11.793)	0.001
Age	0.981 (0.910–1.058)	0.620
Sex	1.442 (0.4733–4.397)	0.520
BODE Index	1.588 (1.130–2.323)	0.008
SGRQ		
Exacerbator Phenotype	3.717 (1.408–9.813)	0.008
Age	1.000 (0.924–1.082)	0.997
Sex	2.606 (0.804–8.450)	0.110
BODE Index	1.814 (1.254–2.623)	0.002

Data are expressed as odds ratio (95% confidence interval).

CAT: COPD Assessment Test; SGRQ: Saint George Respiratory Questionnaire.

**Figure 3:**
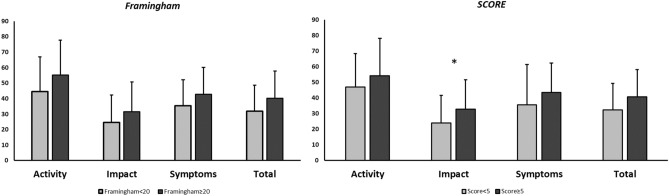
Differences in SGRQ score according to the presence or absence of high CVR in non-exacerbator patients. ***p < 0.05.

**Figure 4:**
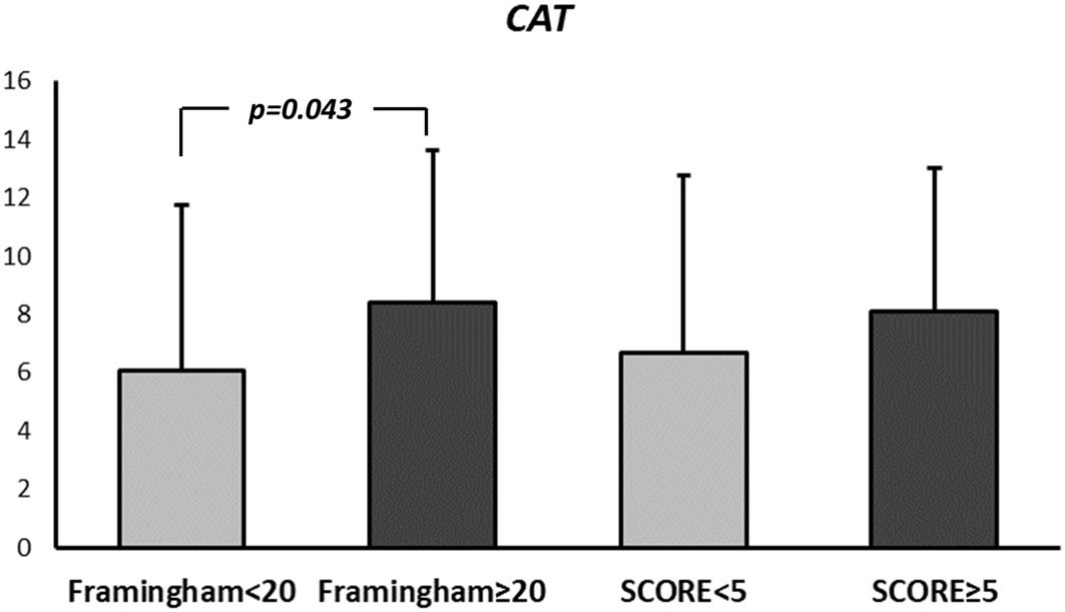
Differences in the CAT questionnaire according to the presence or absence of high CVR in non-exacerbator patients. ***p < 0.05.

When analysing differences in HRQL according to phenotype, but categorising patients according to CVR (high: Framingham scales ≥ 20% and SCORE ≥ 5%), we observed a tendency towards worse HRQL in non-exacerbator patients with a high CVR, which reached statistical significance for the SGRQ impact score on the SCORE scale (24.0 ± 17.7 vs. 32.9 ± 18.7; *p* = 0.039) (Fig. 3). These differences were not observed in exacerbator patients with a high CVR (Table 6). When relating this same aspect to the CAT questionnaire (Fig. 4), a worse HRQL was also observed in non-exacerbator patients with a high CVR, which was significant on the Framingham scale (6.1 ± 5.7 vs 8.41 ± 5.2; *p* = 0.043). These differences were not observed in exacerbator patients (Table 6).

**Table 6:** Variations in SGRQ and CAT scores based on the presence or absence of high cardiovascular risk (Framingham and SCORE) in exacerbator patients.

	Exacerbator					
	Framingham < 20 n = 15	Framingham ≥ 20 n = 32	*P*	Score < 5 n = 16	Score ≥ 5 n = 31	*p*
SGRQ Activity	73.8 ± 21.9	64.3 ± 25.6	0.225	68.5 ± 25.9	66.8 ± 24.4	0.826
SGRQ Impacts	46.2 ± 27.1	41.1 ± 20.8	0.486	45.3 ± 27.8	41.4 ± 20.2	0.578
SGRQ Symptoms	59.1 ± 24.5	56.9 ± 23.2	0.767	59.9 ± 26.4	56.4 ± 21.9	0.627
SGRQ Total	55.1 ± 25.6	49.8 ± 21.4	0.463	53.3 ± 27.3	50.6 ± 20.3	0.703
CAT	13.5 ± 6.6	11.0 ± 5.9	0.211	13.5 ± 6.2	11.0 ± 6.0	0.197

Data are expressed as mean ± standard deviation.

CAT, COPD assessment test; SGRQ, Saint George Respiratory Questionnaire.

## Discussion

Our study found important differences in the CVR and HRQL between exacerbator and non-exacerbator patients, and particularly among patients younger than 65 years and those who had more hospitalisations. The patients with an exacerbator phenotype and the more severe patients experienced a greater worsening in overall HRQL than those with a non-exacerbator phenotype. In relation to the association between CVR and HRQL in our study, it was notable that the non-exacerbator patients with a high CVR were those with a worse HRQL. This finding has not previously been reported.

Our results on HRQL in patients with COPD exacerbations are largely consistent with the literature. Various studies have found an independent association of HRQL, determined by the SF-36 questionnaire, and the risk of hospitalisation and mortality in COPD patients^10,40^. In this study, the SF-36 variables related to physical aspects (pain, physical functioning and general health perception) were the most affected in the exacerbator phenotype. Furthermore, among those patients admitted during the previous year, there was a tendency towards worse values related to physical functioning and general health perception, further highlighting the importance of exacerbations and, therefore, the impact of lack of control of the disease on HRQL^38^.

The correlation between CAT and SGRQ is very good according to the literature, a circumstance confirmed in our study with a correlation in figures very similar to those described^39^. A study with patients with lung function characteristics very similar to ours found higher CAT values for both the exacerbator and the non-exacerbator groups and higher differences between the two phenotypes^41^.

This study confirms with multivariate analysis that patients with exacerbations have worse HRQL regardless of sex and age, as well as the fact that more severe patients (with worse BODE) have worse HRQL. Accordingly, the relevance of controlling the HRQL of our patients is as important as monitoring their lung function or exercise capacity and improving it is a fundamental objective of their treatment, an aspect currently considered in all clinical management guidelines in COPD^1,2^. However, when HRQL was analysed according to CVR, higher CVR values were related with worse HRQL in non-exacerbator patients, a finding not seen in the patients with more frequent exacerbations, whose HRQL was conditioned by their exacerbations.

A recent Canadian cohort study compared healthy people with people with pathological spirometry or in the PRISm (preserved ratio impaired spirometry) range, finding a significantly higher prevalence of CVD among those with impaired spirometry and COPD compared to those with normal spirometry. The prevalence of CVD was significantly higher in participants with PRISm results and GOLD stage II COPD, leading them to conclude that people with poor spirometry, especially those with moderate COPD and patients with PRISm, have a higher incidence of CVD compared to those with normal spirometry, such that having COPD increases the risk of developing CVD^42^. A previous study by our group analysed CVR in the total sample of COPD patients and found elevated values in all the scales used, both in exacerbator and non-exacerbator patients, but there were no significant differences according to clinical phenotypes^43^.

In our study, we found differences in HRQL between non-exacerbator patients with high CVR according to the Framingham (≥ 20%) and SCORE (≥ 5%) scales compared to those with no CVR. Although there are no studies in this regard, this could be due to the impact of CVD on HRQL^44^ and the finding of higher BP levels in our non-exacerbator population with high CVR. A Spanish study applied the SF-36 test to measure the quality of life and related it to CVR using the SCORE and European Society of Hypertension (ESH) tables. It was observed that in the SF-36 subscales “physical functioning” and “health change”, significantly higher values were obtained in subjects with a low-moderate risk SCORE (< 5%) compared to those with a high-risk SCORE (> 5%). In the analysis between hypertension and quality of life (SF-36) those without hypertension had higher scores on all subscales^45,46^. A meta-analysis of 20 observational studies concluded that hypertensive subjects have poorer levels of HRQL than non-hypertensive subjects^47^. In this regard, studies have linked a worsening HRQL with increasing comorbidities in COPD patients. Van Manen et al.^48^ showed that three or more comorbidities correlated better with HRQL scores than with FEV1 or dyspnoea measured by mMRC. Putcha et al. investigated the impact of comorbidities on HRQL, such that for each additional comorbidity, the odds of a deterioration in quality of life increased by 43%. The most prevalent comorbidities, such as heart failure, diabetes or osteoporosis, were individually associated with a significant decrease in quality of life score, adjusted for age, sex, race and other comorbidities^49–52^.

Currently, the analysis of cardiovascular risk factors (CVRF) has great value in defining a common strategy and preventive measures to avoid, from an early age, CV worsening in our COPD patients, so that a better HRQL is associated with a lower CVR^53^. It is important to recognise and treat CVD and the CVRF (like smoking, cholesterol and hypertension) as soon as possible. The adequate treatment of hypertension^54^, diabetes or dyslipidaemia, according to the level of estimated CVR, can contribute to improving HRQL in that it reduces the associated complications^5,6,55^. The GOLD strategy document^1^ establishes that the presence of comorbidities should not, in general, alter the treatment of COPD and that the comorbidities should be treated following usual guidelines, independently of the presence of COPD. Other specialities are aware of the importance of identifying and treating the key comorbidities. For example, the *American Diabetes Association* guidelines on diabetes recognise the need to go beyond just the glycaemia, highlighting the importance of efficient control of hypertension in patients with diabetes^56^.

One of the most notable limitations of our study concerns the fact that the sample was selected from a specific COPD clinic with the patients in advanced stages of the disease, which limits the extrapolation of the results to all COPD patients. Another concern is that the application of any CVR scale has limitations for estimating the overall CVR at the individual level, as its accuracy does not exceed 60%. However, we have used several different scales to attempt to obtain an estimate of CVR as broad and reliable as possible. The sample size may have been insufficient, as we could have obtained greater differences between the two phenotypes with a larger population. Nor did we have a control group of smokers without airway obstruction, which makes it difficult to analyse the results in aspects such as CVR and HRQL, which may be attributable either to smoking or to a direct consequence of the disease. This study is nevertheless relevant as it is one of the few studies to examine CVRF, CVR and quality of life in patients with COPD. Judging by the results of the study, it is already known that exacerbators have a worse quality of life independently of their CVR, but it was the non-exacerbators with a high CVR (poorly studied to date) who have their quality of life restricted and in whom we could undertake an early preventive intervention.

In conclusion, non-exacerbator patients, hypertense and with a high CVR, showed a worse quality of life as measured by the most used HRQL questionnaires. These findings could allow us to control CVRFs (hypertension) more strictly in non-exacerbator COPD patients, so that we can improve their quality of life with certain interventions aimed at reversing a possible worse prognosis.

## Data Availability

Date are available on request due to privacy restrictions. The data presented in this study are available on request from the corresponding author. In compliance with Spanish Organic Law 15/1999, the data are not publicly available.
